# The role of irrational beliefs and motivation regulation in worker mental health and work engagement: A latent profile analysis

**DOI:** 10.1371/journal.pone.0272987

**Published:** 2022-08-15

**Authors:** Martin Turner, Anthony Miller, Hope Youngs

**Affiliations:** 1 Department of Psychology, Manchester Metropolitan University, Manchester, United Kingdom; 2 Health Science and Wellbeing, Staffordshire University, Stoke-on-Trent, United Kingdom; Universiti Pertahanan Nasional Malaysia, MALAYSIA

## Abstract

Research concerning rational emotive behaviour therapy (REBT) and autonomous and controlled motivation within athletic settings is burgeoning. It is proposed that irrational beliefs (i.e., illogical, rigid, and extreme) together with controlled forms of motivation, can determine psychological well-being and physical health in these contexts. For example, research indicates that extreme negative self-evaluation (i.e., self-depreciation) is related to more controlled (less autonomous) motivation regulation, which may underpin poorer health. Though, research is yet to understand the concomitant influence of both irrational beliefs and motivation regulation on work related variables such as presenteeism, persistence and turnover intention, as well as non-work-related variables such as life satisfaction and mental-wellbeing. The present two study paper examines the latent profile structure of irrational beliefs and motivation regulation, and how these latent profiles relate to health and work-related variables. Across studies 1 and 2, results indicated a two-class profile whereby class 1 is characterised by low irrational beliefs and high self-determined motivation (*low irrational engagement*), and class 2 is characterised by high irrational beliefs and low self-determined motivation (*high irrational engagement*). Those in Class 2 reported poorer life satisfaction, persistence, and presenteeism, as well as greater depression, anxiety, stress, intention to quit, and absenteeism than those in class 1. Thus, findings indicate that poorer work and health outcomes are associated with greater irrational work engagement. The findings are discussed in relation to the practical implications for occupational workers.

## Introduction

Modern human beings have selected the name Homosapien for our species. Homo is Latin for ’human’, and sapien is Latin for ’wise’, ’astute’, or ‘judicious’, and human beings are classically considered to be ‘the rational animal’ (Aristotle Metaphysics [1]). However, despite this favourable nomenclature, human beings can of course demonstrably operate unwisely, non-astutely, injudiciously, and irrationally. Oscar Wilde writes in his book The Picture of Dorian Gray that “Man is many things, but he is not rational” [2] and Bertrand Russell [3] noted that, “[I]t has been said that man is a rational animal. All my life I have been searching for evidence which could support this”. Clearly, human beings frequently and ubiquitously think and act in ways that are irrational, and Albert Ellis [4] who pioneered the cognitive-behavioural psychotherapy movement of the 1950s and 1960s makes a point of listing 259 examples of human irrationality. Human beings are capable of rationality, in part because they are capable of irrationality, unlike other animals who can only be arational [5]. But despite our rational capabilities, the deployment of our rationality is not a fixed matter and is not to be assumed. It is perhaps more accurate to suggest that, as John McDowell [6] asserts, we possess a potentiality for rationality which does not imply actuality. Society plays an important role in our rationality [7] and to move from potential rationality to actual rationality, we must engage in a process of initiation into a social practice, such as education [8].

One prominent approach that is proposed to help individuals to develop and strengthen their rationality is rational emotive behaviour therapy (REBT [9]), which is a second-wave cognitive-behavioural psychotherapy (CBT). In REBT rationality is captured in our beliefs about oneself, the world, and other people. Rational beliefs are beliefs that are scientifically warranted, flexible, non-extreme, and usually underpin human survival and fulfilment, at least more so than irrational beliefs which are unscientific, rigid, and absolutistic [7, 10, 11]. In other words, in REBT rational means self-helping and irrational means self-defeating, and as such the chief aims of REBT is to help people weaken their irrational beliefs and strengthen their rational beliefs [12].

Helpfully, in REBT rational and irrational beliefs are captured in four core ideas respectively. Rational beliefs include preferences (“I want to succeed, but it does not mean I must”), anti-awfulizing (“it is bad to fail, but not awful”), frustration tolerance (“it is difficult to not succeed, but I can tolerate it”), and unconditional acceptance (“failing does not make me a complete failure, it just shows that I am an imperfect human being”). In contrast, irrational beliefs include demandingness (“I want to succeed, and therefore I must”), awfulizing (e.g., “it is not just bad to fail, it is awful”), frustration intolerance (e.g., “it is not just difficult to not succeed, it is intolerable”), and depreciation (e.g., “when I fail, it means that I am a complete failure”). Psychological ill-being is underpinned by irrational beliefs, whereas psychological well-being is underpinned by rational beliefs [13]. A meta-analysis of 83 studies (16,110 participants) reported medium effect size associations between irrational beliefs and symptoms of depression, anxiety, anger, distress, and guilt [14]. Importantly, the association between irrational beliefs and mental health was stronger when a stressful event was present (e.g., job stress) than when it was absent. This association is likely to occur due to irrational beliefs (potentially) activating the ventromedial prefrontal cortex, being responsible for dysfunctional emotions and maladaptive behaviours [15, 16]. Specifically, irrational beliefs may activate the anterior/posterior subregions of the ventromedial prefrontal cortex in processing of safety-threat information, producing sustained physiological responses (i.e., stress) [15], contributing to the biological inability to adapt to the demands of the stressful situation [17].

Given the stressful nature of holding irrational beliefs, it is unsurprising that the assessment of irrational beliefs is possible in occupational samples [18]. Whilst the risks of holding irrational beliefs for psychological health and wellbeing is consistently reported in general [14] and athletic populations[19–21], little is known about the relationship between irrational beliefs and workplace well-being and productivity.

In the research that has drawn links between irrational beliefs and poorer mental health, a range of contributing factors have been proposed to help explain this connection. For example, automatic thoughts (e.g., situational inferences [22]), maladaptive schemas (e.g., pervasive self-defeating themes [23]), and threat evaluations (e.g., situational perception of future harm [24]) have been proposed as important factors that might co-occur alongside irrational beliefs to deleteriously predict mental health [21, 25, 26]. Aside from these cognitive-behavioural factors, recent research [27] has indicated the potential alignment of irrational beliefs with the humanistic and organismic perspective offered within self-determination theory (SDT [28, 29]). SDT is an empirically based, organismic theory of human behaviour and personality development that is centrally concerned with human flourishing [30]. At the psychological level SDT differentiates types of motivation along a continuum from controlled to autonomous, which is articulated with one of the sub-theories of SDT; Organismic Integration Theory (OIT [29]).

In OIT, motivation is represented across a continuum of six regulation types from less self-determined (more controlled) to more self-determined (more autonomous); intrinsic motivation (behaviour undertaken for its own sake in the absence of rewards), integrated regulation (behaviour considered personally important but also congruent with other life goals, objectives and needs), identified regulation (behaviour considered worthwhile and important), introjected regulation (behaviour performed to feel worthy or to avoid feelings of guilt or shame), external regulation (behaviour controlled by external forces such as rewards or punishment), and amotivation (lack of intention to enact behavior [30]). External regulation and introjected regulation are considered to be controlling (or low self-determined) forms of motivation and are associated with maladaptive outcomes including low levels of persistence, negative affect, and poor performance on heuristic activities [28]. In contrast, more self-determined forms of motivation (intrinsic, integrated and identified regulation) are related to greater effort, engagement, and task persistence and well-being [31]. Further, controlled motivation regulation is related to elevated burnout, and decreased engagement [32], as well as poorer physical and psychological well-being, greater health risk behaviours, burnout at work, low organisational commitment, greater turnover intention, greater work-family conflict, and overall poorer work performance [33–35]. As such, more self-determined (autonomous) motivation is conducive to psychological health [36–39] and as such should be striven for.

Despite emanating from different schools of psychology, irrational beliefs within REBT and the motivation regulation types within OIT conceptually converge somewhat [40]. For example, irrational beliefs reflect self-pressure (“I must succeed in the things I try”) and contingent self-worth (e.g., “I am worthless if I fail”), where the regulation of behavior is reliant upon rigid and dogmatic ideas about how one *should* be achieving. The direction of action by internal pressure and contingent self-worth is akin to more controlled forms of motivation regulation, specifically introjected regulation [36]. In additional, irrational beliefs concerning the view others have about me (e.g., “I must be approved of by important people”, “I must not be looked down upon”) and external recognition of accomplishments (e.g., “I have to be the best worker in my organization”, “I cannot stand being overlooked for employee awards”), place importance on external factors in regulating one’s actions, reflecting more controlled forms of motivation (i.e., external regulation). Importantly, workers who hold irrational beliefs and whose actions are regulated by more controlled forms of motivation, both underpinned by dogmatic self-pressure, contingent self-worth, and a drive to gain approval and reward, are in precarious position when it comes to their mental health and work engagement. They are likely to engage in work because they believe they have to (rather than want to), consider setbacks and vicissitudes to be an indication of their uselessness, and be highly sensitive to failure and negative feedback. Indeed, Wijhe et al. [41] studied workaholism and found that internalized (irrational) external performance standards to protect self-worth was a vulnerability factor for workaholism. In addition, individuals who are extremely depreciating of themselves are unlikely to perceive themselves as being competent or self-efficacious [42], and thus could be more likely to experience amotivation, a form of which is characterized by a felt lack of competence [30].

This conceptual convergence is not merely academic[27, 43]. There is evidence that by reducing irrational beliefs it is possible to encourage more autonomous motivation regulation [44, 45] with downstream improvements in self-efficacy [42] and sleep and wellbeing [46]. The effects of increasing autonomous motivation through reducing irrational beliefs speaks to a potential co-occurrence of irrational beliefs and motivation regulation. Despite the proposed convergence of REBT irrational beliefs and OIT motivation regulation types, research has been small *n* and is yet to study the extent to which these constructs can together indicate psychological health. The potential health risks of irrational beliefs and low self-determined motivation is unknown at present, and the question remains whether and to what extent irrational beliefs and motivation co-occur to influence psychological health. In addition, to our knowledge, research is yet to determine the effects of this REBT-OIT convergence on work engagement. In the current paper, we consider work engagement to be “an active, work-related psychological state that includes perceptions, emotions, and behaviors, with the features of energy and involvement” [47]. To capture work engagement, we utilise a range of measures across two separate studies. In study 1 we assess mental wellbeing and persistence, and in study 2 we measure persistence but expand our assessment to psychological distress (stress anxiety, and depression), procrastination, absenteeism, presenteeism, and intention to quit. In both studies, our chief aim is to examine how irrational beliefs together with motivation regulation relate to markers of engagement.

To achieve the above aim, in the present paper we adopt person-centered profiling methods by employing latent profile analysis (LPA), allowing us to identify subgroups drawn from data regarding irrational beliefs, motivation, and mental health and engagement markers. Behaviour is motivated by multiple different reasons simultaneously, which in the case of motivation can interact [27] to predict behavioural outcomes. LPA with its person-centered approach can provide complex combinations of several REBT and motivation dimensions. Thus, we take a categorical latent variable, or a person-centred (rather than variable-centred), approach [48], to assess whether irrational beliefs and motivation form differentiable latent profiles. We then use these differentiated profiles to test for differences between profiles in outcome variables, specifically psychological wellbeing and persistence in study 1, and psychological distress, procrastination, absenteeism, presenteeism, intention to quit, and persistence in study 2. Understanding factors that could sensitize workers to poorer mental health and work engagement could help to generate effective interventions and programmes designed to promote work health and engagement. Annually, 15.8 million working days (11.5%) are lost to stress, anxiety and depression, affecting workers across all industries [49], costing the U.K. economy £70 billion per year. The estimated cost of mental illness to U.K. employers due to absenteeism, presenteeism, and employee turnover is £26 billion per year [50]. Clearly, the psychological health of employees can impact upon work engagement, and therefore, antecedents to employee psychological ill-health are worthy of investigation.

As it stands, research has demonstrated that both irrational beliefs and self-determined motivation are important for well-being and workplace engagement. However, little is known about how irrational beliefs might relate to self-determined motivation or how these factors might co-occur to indicate well-being and work engagement. Taking into consideration the conceptual [40] and empirical [27] bridging of REBT and OIT, it is hypothesised that individuals participating in the studies will display differentiated profiles, characteristically adaptive (i.e., low irrational beliefs, high autonomous motivation, low amotivation) or maladaptive (i.e., high irrational beliefs; high controlled motivation, high amotivation). We also hypothesise that adaptive profiles will be associated with greater psychological health and work engagement indicators.

## Study 1

### Method

#### Participants

Sample size was determined using the statistical software package GPower 3.0 [51]. To detect the recommended minimum effect size representing a practically significant effect (RMPE) for social science research (*R*^2^ = .04 [*f*^2^ = .043]), with statistical power set at 0.95 and an alpha error probability .05, in a regression-type model with two predictors, the sample size required is 362. In total, 362 employed adults (158 women, 172 men, 32 unreported; *M*_age_ = 42.75, *SD* = 15.39) agreed to participate in the study. All participants were in current employment or self-employed within a private or public-sector organization that had more than ten employees on a part-time or full-time basis. In total, there were 86 occupations within the sample, the most common being administrative staff (*n* = 35), teachers (*n* = 31), IT staff (*n* = 19), retail workers (*n* = 14), carers (*n* = 12), and accountants (*n* = 12). Participants reported an average of 15.39 years’ experience in their current role (*SD* = 11.39 years). A full list of jobs can be found in the S1 File.

#### Measures

*Irrational beliefs*. The irrational performance beliefs inventory (iPBI [18]) is designed for use in performance settings and was validated in occupational samples. The iPBI has 28-items (e.g., “If others think I am no good at what I do, it shows I am worthless”) and a total irrational beliefs score is computed by summing the responses to all items. Responses are made on a five-point scale from 1 (*strongly disagree*) to 5 (*strongly agree*). The iPBI has been shown to have good construct and criterion validity [18] and has also demonstrated good predictive validity [52] and test-retest-reliability [53] across various performance contexts. Robust confirmatory factor analyses provided adequate fit for the theorized four-factor structure of irrational beliefs (χ2 (344) = 834.05, *p* < .001, CFI = .90, TLI = .90, SRMR = .06, RMSEA = .06). Cronbach’s α and McDonalds Omega (ω) for demandingness, awfulizing, frustration intolerance and self-depreciation demonstrated at least acceptable internal consistency (α ≥ .78, ω ≥ .78).

*Motivation*. The revised motivation at work scale (R-MAWS [54]) was used to contextually measure five forms of motivation in SDT; external regulation (six items), introjected regulation (four items), identified regulation (three items), intrinsic motivation (three items) and amotivation (three items). Items were scored on a seven-point scale from 1 (*not at all*) to 7 (*completely*). The R-MAWS has evidenced convergent, discriminant, and predictive validity [54]. Robust confirmatory factor analyses provided questionable fit for the theorized five-factor structure of motivation regulation (χ2 (137) = 630.65, *p* < .001, CFI = .88, TLI = .85, SRMR = .10, RMSEA = .10). Cronbach’s α and McDonalds Omega (ω) for the five forms of motivation demonstrated at least acceptable internal consistency (α ≥ .80, ω ≥ .79).

*Psychological well-being*. Two measures of well-being were included: one assessing mental well-being and the other assessing general life satisfaction. The short Warwick-Edinburgh Mental Well-Being Scale (SWEMWBS [55]) was used to measure psychological well-being and has seven items (e.g., “I’ve been feeling optimistic about the future”) scored on a five-point scale from 1 (*none of the time*) to 5 (*all of the time*). The SWEMWBS has been found to be psychometrically robust across a range of samples [56, 57]. For life satisfaction, the Office for National Statistics [11] subjective well-being questions were used, which has four questions assessing how satisfied people are with life, the extent to which they believe things they do are worthwhile, how happy they felt yesterday, and how anxious they felt yesterday. Participants responded to each item on an 11-point scale from 0 (*not at all*) to 10 (*completely*). One item is reverse-scored, and higher scores indicate greater life satisfaction. Robust confirmatory factor analyses provided adequate fit for the theorized unidimensional structure of well-being (χ2 (14) = 115.91, *p* < .001, CFI = .92, TLI = .88, SRMR = .05, RMSEA = .14) and a good fit for the theorized unidimensional structure of life satisfaction (χ2 (3) = 701.06, *p* < .001, CFI > .95, TLI > .95, SRMR < .04, RMSEA < .06). Cronbach’s α and McDonalds Omega (ω) for well-being and life satisfaction demonstrated at least good internal consistency (α ≥ .88, ω ≥ .88).

*Persistence*. We used the motivational persistence scale [58] to assesses short-term and long-term persistence. Specifically, we measured current purpose pursuing (CPP; ability to persist in short-term tasks despite obstacles– 4-items), and long-term purposes pursuing (LTPP; capacity to sustain long-term actions– 4-items). Responses to each of the 8-items us made on a five-point Likert-scale from 1 (*a very low degree*) to 5 (*a very high degree*). Higher scores indicate greater persistence. Robust confirmatory factor analyses provided a good fit for the theorized two-factor structure of persistence (χ2 (28) = 1042.00, *p* < .001, CFI = .95, TLI = .93, SRMR = .04, RMSEA = .08). Cronbach’s α and McDonalds Omega (ω) for the persistence markers demonstrated at least acceptable internal consistency (α ≥ .74, ω ≥ .74).

#### Procedure

Ethical approval was obtained from Staffordshire University’s research ethics committee prior to data collection. Participants were provided general information about the study requirements and provided digitised informed consent prior to completing the questionnaires. All questionnaires were completed online through an anonymized system. The questionnaires took no longer than fifteen minutes to complete and participants did not receive any compensation for their voluntary participation in the study.

#### Analytic strategy

Latent Profile Analyses (LPA) identified patterns across irrational beliefs and motivation regulation, following Turner et al.’s [27] procedure. The R package (v. 4.0.2) tidyLPA was used to identify latent profiles [59]. Information-theoretic method, and entropy-based criterion were used to help decide on the best-fitting model. This included; Akaike Information Criteria (AIC), the Bayesian Information Criteria (BIC), Sample Adjusted Bayesian Information Criteria (SABIC), Approximate Weight of Evidence (AWE), Classification Likelihood Criterion (CLC), Kullback Information Criterion (KIC) values [60] and entropy values [27]. The meaning of the profiles that emerge are also important [61, 62], thus both statistics and theoretical underpinnings were considered in identifying the best fitting model [61]. An intercorrelation matrix (see Table 1) identified that intercorrelations between variables were below .80 [63].

**Table 1:** Scale reliabilities, descriptive statistics and inter-correlations Study 1.

	Mean +/- SD	1	2	3	4	5	6	7	8	9	10	11	12	
1. Demandingness	25.88 +/- 3.61	-												
2. Awfulizing	24.33 +/- 4.02	.71**	-											
3. Frustration Intolerance	24.51 +/- 4.05	.60**	.69**	-										
4. Depreciation	17.70 +/- 6.23	.25**	.41**	.39**	-									
5. Intrinsic	15.12 +/- 3.68	.15**	.16**	.23**	.01	-								
6. Identified	16.66 +/- 2.95	.27**	.22**	.29**	-.15**	.56**	-							
7. Introjected	19.67 +/- 4.52	.40**	.43**	.41**	.25**	.27**	.47**	-						
8. External	22.05 +/- 4.02	.43**	.38**	.31**	.24**	.31**	.34**	.49**	-					
9. Amotivation	8.93 +/- 5.17	-.05	.06	.01	.51**	-.16**	-.39**	-.003	.11*	-				
10. Well-being	3.70 +/- .68	.10	.02	.08	-.20**	.51**	.33**	.12*	.18**	-.15**	-			
11. Life satisfaction	6.60 +/- 1.64	-.02	-.10	-.02	-.30**	.44**	.27**	.04	.07	-.26**	.70**	-		
12. Long term persistence	3.61 +/- .72	.13*	.18**	.28**	.001	.49**	.39**	.33**	.30**	-.08	.53**	.42**	-	
13. Short term persistence	3.74 +/- .75	.12*	.15**	.27**	-.13*	.44**	.46**	.31**	.24**	-.21**	.53**	.37**	.73**	-

*Note*: *p* ≤ .05*, *p* ≤ .01**

Second, univariate and multivariate analyses of variance (ANOVA and MANOVA respectively) identified whether there was a significant difference in outcome variables between the latent profiles identified. Data-points with *z* scores greater than 3.29 [64], were winsorized whereby extreme values are replaced to reduce the influence of outliers on the data. Overall, 37 cases were winsorized (< .001% [65]).

### Results

#### Latent profile analysis

AIC (5500.82), AWE (6202.54), BIC (5714.86), CLC (5392.19), KIC (5558.82)^1^, SABIC (5540.37), entropy values (.69) and BLRT *p*-values (< .01) were all most reliable within a two profile solution (varying variance and covariance; Model 6: see S3 File).

**Fig 1:**
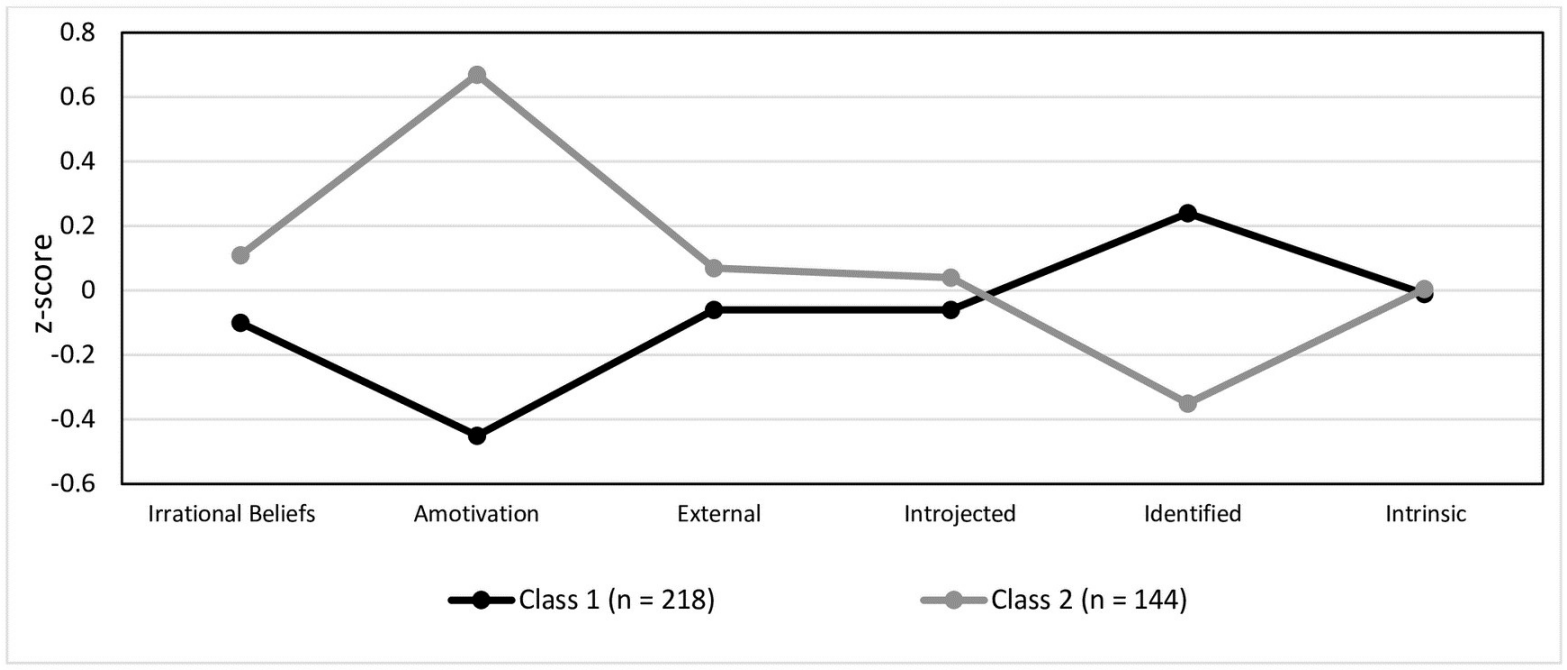
Latent profile analysis. Estimates of the variables for the two latent profile analysis (LPA) classes in Study 1.

Class 1 comprised of 218 participants (60.22%% of the sample; 94 males, 111 females, 13 unreported), Class 2 comprised of 144 participants (39.78% of the sample, 78 males, 47 females, 19 unreported). Those in Class 1 reported lower irrational beliefs, amotivation, and controlled motivation (i.e., external and introjected) relative to Class 2 (Fig 1). In addition, those in Class 1 reported higher autonomous motivation (i.e., identified) than those in Class 2. Differences in intrinsic motivation were minimal (Fig 1).

We evidence two classes, those who hold high irrational beliefs, high amotivation, and high controlled motivation regulation, and low autonomous motivation regulation, (Class 2), and those who hold low irrational beliefs, low amotivation and low controlled motivation regulation, alongside high autonomous motivation regulation (Class 1). As such, Class 2 is characterised by high irrational beliefs and low self-determination, whilst Class 1 is characterised by low irrational beliefs and high self-determination.

#### Well-being and life satisfaction

In understanding whether there is a difference in well-being and life satisfaction between the two classes, ANOVA’s were conducted (Fig 1). There was a non-significant effect of Class on mental-wellbeing (*F*(1, 362) = .09, *p* = .771, *η*^*2*^_*p*_ < .001). There was a significant effect of Class on perceived life satisfaction (*F*(1, 362) = 4.05, *p* = .045, *η*^*2*^_*p*_ = .011). Specifically, those in Class 2 (higher irrational beliefs, predominantly non-self-determined) reported significantly lower life satisfaction than those in Class 1 (lower irrational beliefs, predominantly self-determined).

#### Persistence

In understanding whether there is a difference in persistence between the two classes, a MANOVA was conducted (Fig 1). There was a significant main effect of Class on persistence (Wilks’ *Λ* = .98, *F*(2, 359) = 3.08, *p* = .047, *η*^*2*^_*p*_ = .017). Post hoc analysis revealed that short term persistence was greater in those within Class 1 (lower irrational beliefs, predominantly self-determined) than in Class 2 (*p* = .014). Differences in long term persistence were minimal (*p* > .05).

### Discussion

Results from Study 1 identified that a two-class solution best fit the latent profile structure of irrational beliefs and motivation regulation. Those who reported high irrational beliefs, high amotivation, high controlled motivation regulation, and low autonomous motivation regulation, were likely to report poor life satisfaction and lesser short-term persistence (Class 2). Conversely, individuals who reported low irrational beliefs, low amotivation, and low controlled motivation regulation alongside high autonomous motivation regulation, were likely to report greater life satisfaction and short-term persistence (Class 1). Based on these results, it is evident that a profile characterized by higher irrational beliefs and less self-determined motivation regulation is related to poor life satisfaction and lesser short-term persistence.

In study 2, we use Schmidt’s [66] guidelines to replicate and extend study 1. Schmidt [66] posited that in order to demonstrate the same result as study 1 with a different sample, a modified procedure is required. We adopt psychological ill-being markers (instead of well-being markers), and additional workplace productivity markers. Specifically, in study 2 we shift focus towards psychological distress, and workplace productivity (indicated by markers of procrastination, absenteeism, presenteeism, intention to quit, and persistence). These productivity markers were selected because of their importance for work performance [67, 68] and their established associations with psychological well-being [69]. In addition, research has reported that irrational beliefs relate to procrastination [70, 71], and researchers have predicted that the positive relationship between irrational beliefs and workplace productivity (workaholism) can be explained using SDT [41]. Indeed, one investigation found a positive relationship between workaholism and introjected regulation [72] and one of the more consistent findings in this research area is that stress relates to intention to quit [73]. Irrational beliefs might relate to workplace productivity because the controlling motives that manifest through irrational beliefs lead to greater psychological stress that is detrimental to performance.

## Study 2

### Method

#### Participants

As in Study 1 we aimed to detect the recommended RMPE for social science research (*R*^2^ = .04) and targeted the same sample size of 362 participants. In total, 362 employed adults (*M*_age_ = 43.55, *SD* = 13.51) agreed to participate in the study (183 women, 154 men, 25 unreported sex). All participants were in current employment or were self-employed within a private or public-sector organization that had more than 10 employees. In total, there were 77 occupations within the sample, the most common being IT staff (*n* = 27), retail workers (*n* = 26), administrative staff (*n* = 26), teachers (*n* = 26), checkout operatives (*n* = 12), and accountants (*n* = 12). Participants reported a mean of 14.10 years’ experience in their current role (*SD* = 10.80 years) and were working in the UK at the time of data collection. A full list of jobs can be found in the S2 File.

#### Measures

*Irrational beliefs*. Consistent with Study 1, the iPBI [18] was used to measure irrational beliefs. Robust confirmatory factor analyses provided adequate fit for the theorized four-factor structure of irrational beliefs (χ2 (344) = 1064.72, *p* < .001, CFI = .90, TLI = .90, SRMR = .06, RMSEA = .08). Cronbach’s α and McDonalds Omega (ω) for demandingness, awfulizing, frustration intolerance and self-depreciation demonstrated at least acceptable internal consistency (α ≥ .79, ω ≥ .79).

*Motivation*. Consistent with Study 1, the R-MAWS [54] was used to measure contextual motivation. Robust confirmatory factor analyses provided questionable fit for the theorized five-factor structure of motivation regulation (χ2 (137) = 799.22, *p* < .001, CFI = .86, TLI = .82, SRMR = .11, RMSEA = .12). Cronbach’s α and McDonalds Omega (ω) for the five forms of motivation demonstrated at least acceptable internal consistency (α ≥ .80, ω ≥ .76).

*Psychological distress*. Study 2 used more targeted measures of psychological ill-being in place of general life satisfaction, measuring symptoms of stress, anxiety, depression, anger and curiosity. For stress, we used the perceived stress scale (PSS [74]). The PSS is the most widely used instrument for measuring psychological stress, and captures appraisal of stressful life events over the previous month (e.g., “In the last month, how often have you been upset because of something that happened unexpectedly?”). The scale includes 10 items scored on a five-point scale from 0 (*never*) to 4 (*very often*). The PSS is an easy-to-use questionnaire with established acceptable psychometric properties [75]. Robust confirmatory factor analyses provided a good fit for the theorized unidimensional structure of stress (χ2 (15) = 1447.60, *p* < .001, CFI = .97, TLI = .95, SRMR = .03, RMSEA = .11). Cronbach’s α and McDonalds Omega (ω) for stress was excellent (α = .92, ω = .92).

For anxiety, depression, and anger, we used the trait items from the State-trait personality inventory (STPI [76]). The STPI trait scales include 10-items per subscale. Participants rated their experience of each subscale on a 4-point scale from 1 (*almost never*) to 4 (*almost always*). STPI trait scales have demonstrated high internal consistency coefficients in previous studies ranging from .80 to .96 [76]. Robust confirmatory factor analyses provided questionable fit for the theorized unidimensional structure of anxiety (χ2 (45) = 1388.87, *p* < .001, CFI = .86, TLI = .82, SRMR = .08, RMSEA = .12), anger (χ2 (45) = 1684.95, *p* < .001, CFI = .85, TLI = .81, SRMR = .10, RMSEA = .14), and depression (χ2 (45) = 2271.97, *p* < .001, CFI = .76, TLI = .69, SRMR = .14, RMSEA = .20). That said, Cronbach’s *α* and McDonalds Omega (ω) across subscales demonstrated at least good internal consistency (*α* = .85, ω = .85).

#### Work engagement

*Procrastination*. The procrastination scale [77] is a 20-item measure of procrastination (the action of delaying or postponing something). Participants are asked to indicate how characteristic the 20 statements are of them on a 5-point Likert-scale from 1 (*extremely uncharacteristic*) to 5 (*extremely characteristic*). For example, one item is “I often find myself performing tasks I had intended to do days before”. Of the 20-items, 10-items are reversed-scored (e.g., “I usually make decisions as soon as possible”). Higher scores reflect greater procrastination. Robust confirmatory factor analyses provided adequate fit for the unidimensional structure of procrastination (χ2 (45) = 1586.11, *p* < .001, CFI = .90, TLI = .87, SRMR = .06, RMSEA = .11). Cronbach’s α and McDonalds Omega (ω) for procrastination demonstrated good internal consistency (α = .86, ω = .97).

*Intention to quit*. The three-item intention to turnover scale (ITS [78]) was used to indicate participant turnover intentions. Participants are asked to rate the extent to which they agree with the three statements on a five-point scale ranging from 1 (*strongly disagree*) to 5 (*strongly agree*). One item is “I frequently think of quitting my job”, and the reverse scored item is “If I have my own way, I will be working for my current employer one year from now.” A higher score indicates a greater intention to quit. Robust confirmatory factor analyses provided good fit for the unidimensional structure of intention to quit (χ2 (3) = 341.50, *p* < .001, CFI > .95, TLI > .95, SRMR < .04, RMSEA < .06). Cronbach’s α and McDonalds Omega (ω) for intention to quit demonstrated less than adequate internal consistency (α < .70, ω < .70).

*Absenteeism and presenteeism*. The absenteeism and presenteeism questions of the health and work performance questionnaire (HPQ [79]) was used to measure absenteeism and presenteeism. For absenteeism, participants are asked to indicate how many hours their employer expects them to work in a typical seven-day week, and then how many hours they actually worked in the past 28 days. The hours they are expected to work in seven days are multiplied by four, and then the actual days they worked in the past 28-days are subtracted from that score to compute an absolute absenteeism score. Therefore, absenteeism reflects the number of hours lost per month, with higher scores reflecting greater levels of absenteeism. For presenteeism, participants are asked to indicate their overall job performance on the days they worked during the past 28 days from 0 (*worst performance*) to 10 (*top performance*). Scores are then multiplied by ten to create a percentage score where 0 indicates a total lack of performance and 100 indicates no lack of performance. The HPQ has excellent reliability, validity, and sensitivity to change [79].

*Persistence*. As in study 1, we used the motivational persistence scale [58] to assesses short-term and long-term persistence. Robust confirmatory factor analyses provided a good fit for the theorized two-factor structure of persistence (χ2 (28) = 956.92, *p* < .001, CFI = .95, TLI = .92, SRMR = .04, RMSEA = .09). Cronbach’s α and McDonalds Omega (ω) for the persistence markers demonstrated at least acceptable internal consistency (α ≥ .74, ω ≥ .75).

#### Procedure

Ethical approval was obtained from Staffordshire University’s research ethics committee prior to data collection. Participants were provided general information about the study requirements and provided digitised informed consent prior to completing the questionnaires. All questionnaires were completed online through an anonymized system. The questionnaires took no longer than fifteen-minutes to complete and participants did not receive any compensation for their voluntary participation in the study. There were no outliers in the dataset (no data-points with *z* scores greater than 3.29).

### Results

#### Latent profile analysis

Means, standard deviations and bivariate correlations for measured variables are available in Table 2. AIC (5211.25), AWE (5952.54), BIC (5445.29), CLC (5063.06), KIC (5309.26) [60], SABIC (5300.81), entropy values (.90) and BLRT *p*-values (< .01), were all most reliable within a two profile solution (varying variance and covariance; Model 6: see S4 File).

**Table 2:** Scale reliabilities, descriptive statistics and inter-correlations Study 2.

	Mean +/- SD	1	2	3	4	5	6	7	8	9	10	11	12	13	14	15	16	17	18	19	
1. Demandingness	25.98 +/- 3.83	-																			
2. Awfulizing	24.83 +/- 4.71	.78**	-																		
3. FI	24.65 +/- 4.83	.69**	.70**	-																	
4. Depreciation	17.93 +/- 6.46	.30**	.48**	.47*	-																
5. Intrinsic	14.45 +/- 4.04	.23**	.22**	.19**	.06	-															
6. Identified	16.25 +/- 3.36	.32**	.24**	.28**	-.11*	.48**	-														
7. Introjected	19.13 +/- 4.62	.37**	.39**	.40**	.26**	.40**	.54**	-													
8. External	20.64 +/- 4.87	.46**	.47**	.41**	.32**	.29**	.36**	.57**	-												
9. Amotivation	8.81 +/- 5.28	.04	.13*	.10	.50**	,01	-.26**	.18**	.28**	-											
10. Depression	20.56 +/- 6.36	-.03	.06	.06	.29**	-.17**	-.24**	-.05	-.03	.22**	-										
11. Anxiety	21.64 +/- 5.97	.10	.19**	.19**	.38**	-.11*	-.13*	.10	.12*	.25**	.84**	-									
12. Stress	1.62 +/- .76	.10	.19**	.21**	.39**	-.13*	-.10	.11*	.12*	.29**	.48**	.55**	-								
13. Anger	22.88 +/- 6.58	.26**	.34**	.30**	.38**	.08	.02	.22**	.30**	.27**	.47**	.60**	.35**	-							
14. Procrastination	2.66 +/- .46	.03	.10	.10	.43**	-.10	-.34**	-.004	.14**	.45**	.35**	.35**	.40**	.34**	-						
15. Intent to quit	3.00 +/- .70	.08	.09	.11*	.23**	-.13*	-.03	.10	.15**	.34**	.22**	.26**	.32**	.24**	.21**	-					
16. Presenteeism	1.06 +/- .17	-.06	-.11*	-.10	-.16**	.02	.002	-.09	-.08	-.10	-.12*	-.17**	-.12*	-.09	-.08	-.08	-				
17. Absenteeism	.17 +/- .49	.14**	.12*	.07	.20**	.11*	-.04	.10	,04	.29**	.05	.07	.06	.16**	.03	.06	-.04	-			
18. Performance	7.42 +/- 2.46	.01	-.05	-.03	-.22**	.13*	.19**	-.02	-.03	-.30**	-.24**	-.20**	-.25**	-.20**	-.21**	-.20**	.15**	-.33**	-		
19. LT persistence	3.66 +/- .69	.27**	.22**	.25**	.05	.54**	.49**	.44**	.37**	-.01	-.23**	-.15**	-.07	.06	-.16**	.03	-.02	.05	.10	-	
20. ST persistence	3.80 +/- .66	.23**	.15**	.25**	-.13*	.44**	.57**	.40**	.25**	-.19**	-.28**	-.18**	-.17**	.04	-.36**	-.01	.06	.08	.17**	.68**	-

*Note*: FI = Frustration Intolerance, ST = Short term, LT = Long term, *p* ≤ .05*, *p* ≤ .01**

**Fig 2:**
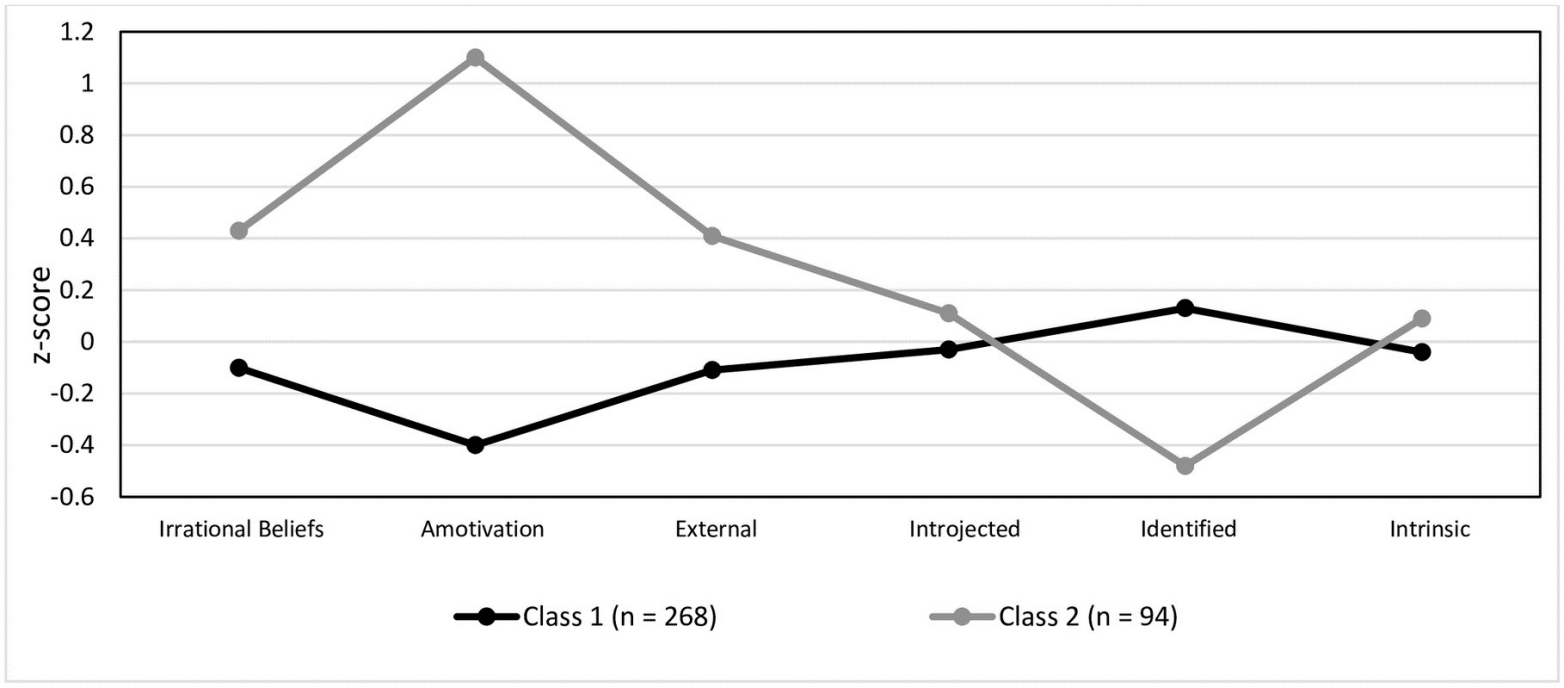
Latent profile analysis. Estimates of the variables for the two latent profile analysis (LPA) classes in Study 2.

Class 1 comprised of 268 participants (74.03% of the sample; 115 males, 137 females, 16 unreported), Class 2 comprised of 94 participants (25.97% of the sample, 39 males, 46 females, 9 unreported). Those in Class 1 reported lower irrational beliefs, amotivation, and controlled motivation (i.e., external) relative to Class 2 (Fig 2). In addition, those in Class 1 reported higher autonomous motivation (i.e., identified) than those in Class 2. Differences in introjected regulation and intrinsic motivation were minimal (Fig 2).

The patterns evidence two classes, those who hold high irrational beliefs, high amotivation, and high controlled motivation regulation, and low autonomous motivation regulation (Class 2), and those who hold low irrational beliefs, low amotivation and low controlled motivation regulation, alongside high autonomous motivation regulation (Class 1). As such, Class 1 is characterised by low irrational beliefs and high self-determination, whilst Class 2 is characterised by high irrational beliefs and low self-determination.

#### Psychological distress

In understanding whether there is a difference in psychological distress between the two classes, MANOVA examined possible differences in symptoms of depression, anxiety, stress, and anger (Fig 2). There was a significant main effect of Class on depression, anxiety, and stress (Wilks’ *Λ* = .97, *F*(4, 357) = 2.49, *p* = .04, *η*^*2*^_*p*_ = .027). Follow up comparisons identified that depression, anxiety, and stress were significantly higher in Class 2 (higher irrational beliefs, predominantly non-self-determined) than in Class 1 (lower irrational beliefs, predominantly self-determined; *p* < .05). Those in Class 2 also reported close-to-significantly higher anger than those in Class 1 (*p* = .06).

#### Work engagement

In understanding whether there is a difference in procrastination, intention to quit, relative presenteeism and relative absenteeism between the two classes, ANOVAs were conducted (Fig 2).

There was a significant effect of Class on procrastination (*F*(1, 360) = 42.09, *p* < .001, *η*^*2*^_*p*_ = .11), intention to quit (*F*(1, 360) = 19.91, *p* < .001, *η*^*2*^_*p*_ = .05), relative presenteeism (typical hours working; *F*(1, 360) = 4.58, *p* = .033, *η*^*2*^_*p*_ = .013; typical job performance; *F*(1, 357) = 25.26, *p* < .001, *η*^*2*^_*p*_ = .066), relative absenteeism (*F*(1, 360) = 15.24, *p* < .001, *η*^*2*^_*p*_ = .04), and short term persistence (*F*(1, 360) = 12.01, *p* = .001, *η*^*2*^_*p*_ = .03). Namely, those in Class 2 (higher irrational beliefs, predominantly non-self-determined) reported greater procrastination, intention to quit, absenteeism, and lower presenteeism and short term-persistence than Class 1 (lower irrational beliefs, predominantly self-determined).

### Discussion

Results from Study 2 identified that a two-class solution best fit the latent profile structure of irrational beliefs and motivation. Those who reported high irrational beliefs, high amotivation, and high controlled motivation regulation, were likely to report greater depression, anxiety, stress, and anger (Class 2). In addition, those in class 2 were also more likely to report greater procrastination, intention to quit and absenteeism, as well as lower presenteeism and short term-persistence. In contrast, participants who reported low irrational beliefs, low amotivation, and low controlled motivation regulation, were likely to report lower depression, anxiety, stress, and anger, as well as less procrastination, intention to quit and absenteeism, and greater presenteeism and short term-persistence (Class 1). Based on these results, it is evident that a profile characterized by high irrational beliefs and low self-determined motivation regulation is related to greater psychological distress and poorer work engagement.

## General discussion

The current paper offers empirical convergence of REBT and OIT, identifying the potential consequences of a combination of both irrational beliefs, and less self-determined motivation on work engagement and wellbeing. Broadly, the results indicate two-class profiles characterised by different levels of irrational beliefs and self-determined motivation. In one class, which we will call *high irrational engagement*, individuals report higher irrational beliefs and lower self-determined motivation, and a second class, which we will call *low irrational engagement*, individuals report lower irrational beliefs and higher self-determined motivation. Compared to the *low irrational engagement* profile, the *high irrational engagement* indicated poorer life satisfaction, persistence, presenteeism, absenteeism, and higher intentions to quit, as well as greater symptoms of depression, anxiety, stress, and anger. In other words, organisational workers who hold irrational beliefs about their work, whose engagement in tasks are driven by reward seeking, avoiding punishments, and guilt, or are not motivated to engage at all (i.e., *high irrational engagement*), are likely to report poorer psychological wellbeing and poorer work engagement.

The findings of the present paper offer some support for previously hypothesised convergences between REBT and OIT [27]. Specifically, irrational beliefs and motivation regulation types were related to one another such that high irrational beliefs were more strongly associated with more controlling forms of motivation. This was evidenced by the differentiated profiles that emerged in the LPAs. In addition to offering some evidence of theoretical convergence, the current paper adds to and builds upon existing research that indicates the potential consequences of such convergence. Whilst past work has indicated that *low irrational engagement* is advantageous for the mental and physical health of athletes and exercise participants [27], the current study offers supporting evidence in an occupational sample but also extends findings beyond wellbeing. That is, in the current study we include engagement markers of persistence, persistence, absenteeism, and intentions to quit, which offer behavioural indicators that extend the potential implications of *low irrational engagement* beyond health. In a previous study, Wijhe et al. [41] evidenced that internalizing external performance standards (an irrational belief) to protect self-worth is likely to lead to workaholism, whilst other irrational beliefs did not associate with workaholism. In explaining this discrepancy, we posit that the influence of irrational beliefs on work engagement and workaholism is conditional, depending on an individuals’ motives. Based on the results of the current paper, it is particularly when irrational beliefs are high, and self-determined motivation is low (i.e., *high irrational engagement*) that work engagement and wellbeing suffers. As such, it is possible that irrational beliefs may influence self-determined motivation such that greater irrationality leads to more controlled forms of motivation regulation, and as a result, work engagement and wellbeing is negatively impacted. However, these causal hypotheses need to be empirically tested, beyond the evidence found in cross-sectional (the current paper) and applied studies [42].

It is important to elucidate psychologically-derived worker profiles that can indicate wellbeing and work engagement so that we can design strategies to improve particularly salient psychological factors. Specifically, as is proffered within REBT, irrational beliefs can be forthrightly weakened in occupational samples with a view to enabling adaptive engagement with ones’ environment [80]. There is also some growing evidence that by weakening irrational beliefs, self-determined motivation can be fostered [42], with beneficial effects upon wellbeing [44]. The reported use of REBT within occupational samples indicates its effectiveness (*d* = -1.14) in reducing worker distress [80]. In a recent study, researchers found that using REBT to decrease irrational beliefs in police officers had beneficial effects on self-determined motivation [81]. Therefore, based in part on the evidence presented in the present study, it might be fruitful to develop work-based programs that target the enhancement of *low irrational engagement*, and dissuade *high irrational engagement*.

It should also be noted that the findings of the current paper are consistent with the theoretical postulations of both REBT and SDT, in that greater irrational beliefs are associated with indicators of poorer wellbeing [14], and work engagement [70], as is lower self-determined motivation [35]. Furthermore, whilst only at a correlational level, the present paper indicates that markers of poor wellbeing are related to markers of poor work engagement, in support of past research [26]. Thus, in order to promote greater work engagement, it seems reasonable to encourage rationality, self-determined motivation, and high wellbeing in workers. Away from the evidence offered in the present paper, and the past research that corroborates it, logically there appears to be little downside in promoting rationality and autonomous motivation regulation (i.e., *low irrational engagement*).

The promotion of *low irrational engagement* is perhaps best achieved through individual and environmental adaptations. The individual can be encouraged to weaken their irrational beliefs about work performance using one-to-one coaching [81]. But of course, the positive effects of this adaptation is limited to the individual in receipt of the coaching (notwithstanding the individual’s proclivity to share what they have learned with others). Therefore, it might be more fruitful and efficient to develop systemic strategies that promulgate rationality and autonomous regulation across work forces. Indeed, there is evidence from sport research that soccer coaches can encourage rational engagement in important tasks [82] and that group educational programs can weaken irrational beliefs and simultaneously enhance self-determined motivation [46]. For occupational settings, some suggest that, “REBT is the most business friendly school of psychology when it comes to helping executives, managers, and firms solve people problems, enhance productivity, and help senior people become more effective leaders and managers” [83]. Indeed, REBT is particularly useful in times of difficulty because it enables the individual to exercise some responsibility over their emotion and behaviour despite duress. Turner and Barker [84] delivered two 4-hour intensive REBT workshops to a group of professionals (*n* = 11) working within a blue-chip organisation, but who were being made redundant. Some participants reported motivational increments alongside weakened irrational beliefs. In addition, David and Matu [83] implemented REBT for telecommunications managerial staff who had been informed that the factory would be closing. Despite significant distress at the prospect of losing their jobs, through REBT the managers were able to limit the maladaptive expression of this distress by being able to better control their dysfunctional negative emotions.

### Limitations and future directions

The findings of the current paper need to be viewed through a critical lens. Whilst the present paper provides some evidence of the disadvantages of *high irrational engagement* across two separate occupational samples, data were collected using atemporal/cross-sectional methods. Thus, we cannot posit cause-effect relationships between irrational beliefs, motivation regulation, and wellbeing and work engagement outcomes. Future research could undertake experimental research to offer controlled studies that seek to influence irrational beliefs and motivation regulation and measure the resultant effects upon work practices. Also, longitudinal research could be undertaken to assess the extent to which wellbeing and work engagement suffers as a result of *high irrational engagement* across time. One of the main aims of the present paper was to replicate the profiles identified in previous research in sport and exercise domains [27], and as such, a single-timepoint cross sectional approach was taken. But future research could apply research using cross-lagged auto-regression or latent profile transitional analyses [85] with longitudinal data in understanding temporal dynamics of the profiles identified.

Readers should also be aware that the measurements used in the present study were not as psychometrically robust as we would have hoped. Measures of motivation and psychological distress symptomology (anxiety, anger, depression) demonstrated less than acceptable fit indices and therefore future researchers may seek to enhance these measures or use alternative indicators of these target variables. Lastly, the sampling method we employed enabled us to recruit a broad population of workers. However, it might be advantageous to sample specific working populations within particular industries, so that work engagement markers can be more tailored to the participants.

## Conclusions

If we agree with the (self-evident) presupposition that human beings are capable of both rationality and irrationality [4, 6], then the question is whether and to what extent is irrationality a bad thing for human fulfilment. In the present study it is indicated through LPA that participant profiles that are characterised by *high irrational engagement* are associated with poorer wellbeing and work engagement, compared to profiles characterised by *low irrational engagement*. It seems that those reporting high irrational beliefs and less self-determined work motivation are more at risk of poorer psychological wellbeing and poorer work engagement. As such, given the recently found empirical convergence between irrational beliefs and motivation regulation in sport and exercise settings [27, 43], we present grounds for theoretical development within REBT in occupational settings. Rather than REBT and SDT representing two distinct theoretical approaches to work engagement, researchers should explore the convergence of REBT and SDT in order to inform workplace initiatives for the promotion of worker engagement and wellbeing. Organisations should consider employing REBT with a focus on self-determined motivation with workers in order to dissuade *high irrational engagement*. Given that society plays an important role in human rationality [4], the transition from potential rationality to actual rationality can be facilitated by engaging workers in an educational process [8], underpinned by REBT.

## Supporting information

S1 Table Occupation of participants, Study 1. (DOCX)

S2 Table Occupation of participants, Study 2. (DOCX)

S3 Table Fit statistics for latent profile analysis Study 1. (DOCX)

S4 Table Fit statistics for latent profile analysis Study 2. (DOCX)

S1 File (R)

S2 File (CSV)

S3 File (SAV)

S4 File (CSV)

S5 File (SAV)
