# Supplementary material for: The role of irrational beliefs and motivation regulation in worker mental health and work engagement: A latent profile analysis
Source: PLoS One. 2022 Aug 15;17(8):e0272987. doi: 10.1371/journal.pone.0272987 (PMC9377577; doi:10.1371/journal.pone.0272987)
Supplement: S1 Table — (DOCX) [file pone.0272987.s001.docx]

**S1 Table. Occupation of participants, study 1**

| **Occupation** | **Frequency** |
| --- | --- |
| Maintenance staff, Vice Principal Director, Accountant  Nurse, Cleaner  Doctor, Computer Programmer  Checkout Operative, Admin Worker  Traffic Warden, IT  Charity, Payroll Manager  Secretary, Customer Services  Shop Assistant, Consultant  Support Worker, Engineer  Driver, Teacher  Analyst, Retail Worker  Information Officer, Carer  Lunchtime supervisor, Civil Services  Scientist, Receptionist  Government worker, Guest Host  Advisor, Office Worker  Barperson, Personal Assistant  Manual Labourer, Stock Controller  Childcare, Pilot  Tax Worker, Cleric  Unemployed, Plumber  Video game developer, Train dispatcher  Coach, Service Manager  Technician, Seamstress  Waiter/Waitress, Supervisor  Casino Inspector, Editor  Dentist, Foreman  Chef, Manufacturing  Warehouse Operative, Data Quality Officer  Factory Worker, Postal Services  Operations Manager, Contracts  Lawyer, Security  Health and Safety Professional, Project Manager  Therapist, Banker  Business person, Librarian  Insurance worker, Facilities Manager  Bin Man, Pharmacy Assistant  Telecommunications, Public Relations  Social Media Coordinator, Porter  Chauffeur, Marketing  Logistics, Development Coordinator  Interpreter, Architect  Undisclosed | 3, 1  6, 12  7, 9  5, 3  7, 35  1, 19  2, 1  2, 6  5, 5  3, 9  7, 31  3, 14  2, 12  1, 6  3, 4  1, 1  3, 6  2, 1  8, 1  1, 1  2, 1  2, 3  1, 1  1, 1  4, 1  3, 1  1, 2  1, 1  1, 2  4, 2  3, 2  1, 2  1, 4  1, 2  1, 1  1, 3  1, 2  1, 1  1, 1  1, 1  1, 1  1, 1  1, 1  41 |
