# Supplementary material for: The role of irrational beliefs and motivation regulation in worker mental health and work engagement: A latent profile analysis
Source: PLoS One. 2022 Aug 15;17(8):e0272987. doi: 10.1371/journal.pone.0272987 (PMC9377577; doi:10.1371/journal.pone.0272987)
Supplement: S2 Table — (DOCX) [file pone.0272987.s002.docx]

**S2 Table. Occupation of participants, study 2**

| **Occupation** | **Frequency** |
| --- | --- |
| Retail Worker, Admin Worker  Cleaner, Driver  Supervisor, IT  Teacher, Warehouse Operative  Carer, Business person  Production, Manufacturing  Student, Checkout Operative  Auditor, Accountant  Barperson, Alarms Systems  Human Resources, Seamstress  Engineer, Lecturer  Dustman, Retired  Legal Services Worker, Director  Analyst, Nurse  Enumerator, Hospitality  Housewife, Factory Worker  Zookeeper, Youth Worker  Consultant, Finance Director  Government worker, Invigilator  Librarian, Chef  Postal Services, Services  Dentist, Receptionist  Manual Labourer, Leisure worker  Train dispatcher, Environmental Services Worker  Logistics, Telecommunications  Distribution, Housing  Scientist, Security  Hairdresser, Hotel  Stock planner, Health and Safety Professional  Podiatrist, Electrician  Travel Worker, Charity  Paediatrician, Optometrist  Haematology Assistant, Doctor  Pharmacy Assistant, Plumber  Paramedic, Coach  Arts Curator, Vicar  Banker, Trainer  Radiographer, Personal Assistant  Events Coordinator, Undisclosed | 26, 26  4, 8  1, 27  26, 4  13, 4  1, 2  2, 12  1, 12  1, 1  4, 2  7, 4  1, 2  6, 6  4, 5  1, 2  2, 2  1, 1  1, 3  6, 1  4, 1  1, 5  2, 6  9, 1  1, 1  3, 7  3, 3  1, 6  1, 1  2, 1  1, 1  1, 3  1, 1  1, 4  1, 1  1, 1  1, 1  1, 1  1, 1  1, 47 |
