# Supplementary material for: The role of irrational beliefs and motivation regulation in worker mental health and work engagement: A latent profile analysis
Source: PLoS One. 2022 Aug 15;17(8):e0272987. doi: 10.1371/journal.pone.0272987 (PMC9377577; doi:10.1371/journal.pone.0272987)
Supplement: S3 Table — (DOCX) [file pone.0272987.s003.docx]

**S3 Table. Fit statistics for latent profile analysis Study 1**

|  |  | AIC | BIC | AWE | CLC | KIC | SABIC | Entropy | BLRT *p*-value |
| --- | --- | --- | --- | --- | --- | --- | --- | --- | --- |
| Model 1 | 1 Class | 6194.69 | 6241.39 | 6346.09 | 6172.69 | 6209.69 | 6203.32 | 1 |  |
| Model 1 | 2 Classes | 5921.31 | 5995.25 | 6162.72 | 5884.79 | 5943.31 | 5934.97 | .73 | < .01 |
| Model 1 | 3 Classes | 5795.76 | 5896.94 | 6126.43 | 5745.45 | 5824.76 | 5814.45 | .83 | < .01 |
| Model 1 | 4 Classes | 5669.27 | 5797.70 | 6089.50 | 5604.89 | 5705.27 | 5693.00 | .80 | < .01 |
| Model 1 | 5 Classes | 5631.95 | 5787.61 | 6141.65 | 5553.58 | 5674.95 | 5660.71 | .80 | < .01 |
| Model 1 | 6 Classes | 5590.02 | 5772.93 | 6189.18 | 5497.68 | 5640.02 | 5623.82 | .81 | < .01 |
| Model 2 | 1 Class | 6186.02 | 6232.72 | 6337.42 | 6164.02 | 6201.02 | 6194.65 |  |  |
| Model 2 | 2 Classes | 5909.10 | 6006.39 | 6227.29 | 5860.50 | 5937.10 | 5927.08 |  |  |
| Model 2 | 3 Classes |  |  |  |  |  |  |  |  |
| Model 2 | 4 Classes |  |  |  |  |  |  |  |  |
| Model 2 | 5 Classes |  |  |  |  |  |  |  |  |
| Model 2 | 6 Classes |  |  |  |  |  |  |  |  |
| Model 3 | 1 Class | 5662.77 | 5767.84 | 6005.92 | 5610.77 | 5692.77 | 5682.19 | 1 |  |
| Model 3 | 2 Classes | 5628.41 | 5760.73 | 6061.46 | 5561.99 | 5665.41 | 5652.86 | .78 | < .01 |
| Model 3 | 3 Classes | 5520.85 | 5680.41 | 6043.29 | 5440.52 | 5564.85 | 5550.33 | .84 | < .01 |
| Model 3 | 4 Classes | 5502.51 | 5689.31 | 6114.49 | 5408.12 | 5553.51 | 5537.03 | .82 | < .01 |
| Model 3 | 5 Classes | 5499.44 | 5713.48 | 6201.01 | 5390.95 | 5557.44 | 5538.99 | .72 | < .01 |
| Model 3 | 6 Classes | 5485.79 | 5727.07 | 6276.78 | 5383.36 | 5550.79 | 5530.37 | .71 | < .01 |
| Model 6 | 1 Class | 5649.41 | 5754.48 | 5992.56 | 5597.41 | 5679.41 | 5668.83 | 1 |  |
| **Model 6** | **2 Classes** | **5500.82** | **5714.86** | **6202.54** | **5392.19** | **5558.82** | **5540.37** | **.69** | **< .01** |
| Model 6 | 3 Classes |  |  |  |  |  |  |  |  |
| Model 6 | 4 Classes |  |  |  |  |  |  |  |  |
| Model 6 | 5 Classes |  |  |  |  |  |  |  |  |
| Model 6 | 6 Classes |  |  |  |  |  |  |  |  |

Note: Boldface indicates the selected model.

Abbreviations: AIC, Akaike Information Criterion; BIC, Bayesian Information Criterion; AWE, Approximate Weight of Evidence; CLC, Classification Likelihood Criterion; KIC, Kullback Information Criterion; SABIC, Sample Adjusted Bayesian Information Criterion; BLRT, Bootstrap Likelihood Ratio Test. Model 1 = equal variances and covariances fixed to 0; Model 2 = varying variances and covariances fixed to 0; Model 3 = equal variances and covariances; Model 4 and 5 cannot be estimated with the tidyLPA package; Model 6 = varying variances and covariances. For Model 2, the 6-profile version could not be estimated. For model 6, the 6-profile version could not be estimated.
