# Supplementary material for: The role of irrational beliefs and motivation regulation in worker mental health and work engagement: A latent profile analysis
Source: PLoS One. 2022 Aug 15;17(8):e0272987. doi: 10.1371/journal.pone.0272987 (PMC9377577; doi:10.1371/journal.pone.0272987)
Supplement: S4 Table — (DOCX) [file pone.0272987.s004.docx]

**S4 Table. Fit statistics for latent profile analysis Study 2**

|  |  | AIC | BIC | AWE | CLC | KIC | SABIC | Entropy | BLRT *p*-value |
| --- | --- | --- | --- | --- | --- | --- | --- | --- | --- |
| Model 1 | 1 Class | 6194.12 | 6240.86 | 6345.59 | 6172.12 | 6209.12 | 6202.79 | 1 |  |
| Model 1 | 2 Classes | 5912.46 | 5986.45 | 6154.07 | 5875.85 | 5934.47 | 5926.18 | .69 | < .01 |
| Model 1 | 3 Classes | 5723.05 | 5824.30 | 6053.86 | 5672.75 | 5752.05 | 5741.81 | .88 | < .01 |
| Model 1 | 4 Classes | 5681.20 | 5809.72 | 6101.55 | 5616.88 | 5717.20 | 5705.02 | .85 | < .01 |
| Model 1 | 5 Classes | 5576.41 | 5732.19 | 6086.27 | 5498.11 | 5619.41 | 5605.29 | .87 | < .01 |
| Model 1 | 6 Classes | 5493.48 | 5676.52 | 6092.90 | 5401.13 | 5543.48 | 5527.41 | .86 | < .01 |
| Model 2 | 1 Class | 6167.80 | 6214.50 | 6319.20 | 6145.80 | 6182.80 | 6176.43 | 1 |  |
| Model 2 | 2 Classes | 5915.16 | 6012.45 | 6232.77 | 5867.14 | 5943.16 | 5933.15 | .98 | < .01 |
| Model 2 | 3 Classes |  |  |  |  |  |  |  |  |
| Model 2 | 4 Classes |  |  |  |  |  |  |  |  |
| Model 2 | 5 Classes |  |  |  |  |  |  |  |  |
| Model 2 | 6 Classes |  |  |  |  |  |  |  |  |
| Model 3 | 1 Class | 5575.93 | 5681.01 | 5919.08 | 5523.93 | 5605.93 | 5595.35 | 1 |  |
| Model 3 | 2 Classes | 5534.90 | 5667.21 | 5967.91 | 5467.91 | 5468.52 | 5571.90 | .91 | < .01 |
| Model 3 | 3 Classes | 5380.73 | 5540.29 | 5903.08 | 5300.50 | 5424.73 | 5410.22 | .87 | < .01 |
| Model 3 | 4 Classes | 5328.07 | 5514.89 | 5939.95 | 5233.80 | 5379.07 | 5362.59 | .85 | < .01 |
| Model 3 | 5 Classes | 5301.03 | 5515.07 | 6002.45 | 5192.69 | 5359.03 | 5340.58 | .85 | < .01 |
| Model 3 | 6 Classes | 5267.74 | 5509.02 | 6058.58 | 5145.46 | 5332.74 | 5312.32 | .85 | < .01 |
| Model 6 | 1 Class | 5582.36 | 5687.44 | 5925.51 | 5530.36 | 5612.36 | 5601.78 | 1 |  |
| **Model 6** | **2 Classes** | **5211.25** | **5445.29** | **5952.54** | **5063.06** | **5309.26** | **5300.81** | **.90** | < .01 |
| Model 6 | 3 Classes | 5188.54 | 5521.55 | 6247.68 | 5034.41 | 5274.54 | 5248.23 | .89 | < .01 |
| Model 6 | 4 Classes | 5248.04 | 5670.01 | 6655.22 | 5017.80 | 5352.04 | 5317.86 | .88 | < .01 |
| Model 6 | 5 Classes |  |  |  |  |  |  |  |  |
| Model 6 | 6 Classes |  |  |  |  |  |  |  |  |

Note: Boldface indicates the selected model.

Abbreviations: AIC, Akaike Information Criterion; BIC, Bayesian Information Criterion; AWE, Approximate Weight of Evidence; CLC, Classification Likelihood Criterion; KIC, Kullback Information Criterion; BLRT, Bootstrap Likelihood Ratio Test; SABIC, Sample Adjusted Bayesian Information Criterion; BLRT, Bootstrap Likelihood Ratio Test. Model 1 = equal variances and covariances fixed to 0; Model 2 = varying variances and covariances fixed to 0; Model 3 = equal variances and covariances; Model 4 and 5 cannot be estimated with the tidyLPA package; Model 6 = varying variances and covariances.
